# Comparison of different clinical risk scores to predict long-term survival and neurological outcome in adults after cardiac arrest: results from a prospective cohort study

**DOI:** 10.1186/s13613-022-01048-y

**Published:** 2022-08-17

**Authors:** René Blatter, Simon A. Amacher, Chantal Bohren, Christoph Becker, Katharina Beck, Sebastian Gross, Kai Tisljar, Raoul Sutter, Stephan Marsch, Sabina Hunziker

**Affiliations:** 1 Medical Communication and Psychosomatic Medicine, University Hospital Basel, Klingelbergstrasse 23, 4031 Basel, Switzerland; 2 Intensive Care Unit, University Hospital Basel, Basel, Switzerland; 3 Department of Emergency Medicine, University Hospital Basel, Basel, Switzerland; 4 Medical Faculty, University of Basel, Basel, Switzerland

**Keywords:** Cardiac arrest, Cardiopulmonary resuscitation, Long-term survival, CAHP, OHCA, SAPS II, APACHE II

## Abstract

**Background:**

Several scoring systems have been used to predict short-term outcome in patients with out-of-hospital cardiac arrest (OHCA), including the disease-specific OHCA and CAHP (Cardiac Arrest Hospital Prognosis) scores, as well as the general severity-of-illness scores Acute Physiology and Chronic Health Evaluation II (APACHE II) and Simplified Acute Physiology Score II (SAPS II). This study aimed to assess the prognostic performance of these four scores to predict long-term outcomes (≥ 2 years) in adult cardiac arrest patients.

**Methods:**

This is a prospective single-centre cohort study including consecutive cardiac arrest patients admitted to intensive care in a Swiss tertiary academic medical centre. The primary endpoint was 2-year mortality. Secondary endpoints were neurological outcome at 2 years post-arrest assessed by Cerebral Performance Category with CPC 1–2 defined as good and CPC 3–5 as poor neurological outcome, and 6-year mortality.

**Results:**

In 415 patients admitted to intensive care, the 2-year mortality was 58.1%, with 96.7% of survivors showing good neurological outcome. The 6-year mortality was 82.5%. All four scores showed good discriminatory performance for 2-year mortality, with areas under the receiver operating characteristics curve (AUROC) of 0.82, 0.87, 0.83 and 0.81 for the OHCA, CAHP, APACHE II and SAPS II scores. The results were similar for poor neurological outcome at 2 years and 6-year mortality.

**Conclusion:**

This study suggests that two established cardiac arrest-specific scores and two severity-of-illness scores provide good prognostic value to predict long-term outcome after cardiac arrest and thus may help in early goals-of-care discussions.

**Supplementary Information:**

The online version contains supplementary material available at 10.1186/s13613-022-01048-y.

## Background

Cardiac arrest is a global public health challenge and an important cause of premature death [1, 2]. Mortality is high, and survivors frequently carry a significant disease burden due to unfavourable neurological outcomes [3–5]. As a consequence, cardiac arrest leads to significant socio-economic costs [6]. At intensive care unit (ICU) arrival, cardiac arrest patients with return of spontaneous circulation (ROSC) are frequently unconscious and sedated, rendering clinical neurological evaluation difficult [7–9]. Therefore, clinicians have to rely on history and ambiguous clinical and diagnostic findings for early prognostication and adequate counselling of relatives [8, 10]. Although substantial progress has been made in the prognostication of short-term outcomes after cardiac arrest [8, 11–13], prospective data for early prognostication of long-term outcomes (≥ 2 years) after cardiac arrest are scarce [14–16]. Recently, the focus in post-cardiac arrest research has shifted from short-term to long-term outcomes, as highlighted in the 2021 European guidelines for post-resuscitation care and two recently published systematic reviews and a meta-analysis [8, 17, 18]. Reliable prediction of long-term outcomes is of great importance because a change in the level of neurological functioning can still occur months after hospital discharge [8, 13]. In many cases, patients and relatives would agree to limiting therapeutic efforts in the light of poor prognosis, foreseeable poor quality of life or high risk of physical or mental disability [19, 20]. There is a wide consensus among health care professionals that life-sustaining interventions should only be used if they are consistent with the patient’s values and goals [21]. Therefore, prediction of long-term outcomes might provide important additional information to guide early discussions about goals-of-care and the extent of therapeutic effort.

In the past 15 years, several clinical risk scores have been developed and validated specifically for early prognostication after cardiac arrest [22, 23]. Still, only a few have been adequately validated in independent cohorts [22]. In 2006, the Out-of-Hospital Cardiac Arrest (OHCA) score was developed, which relies on five clinical (no-flow and low-flow interval, initial rhythm) and laboratory parameters (creatinine, lactate) available at ICU admission [11]. The Cardiac Arrest Hospital Prognosis (CAHP) score was developed in 2016 and includes additional information regarding resuscitation measures (location of cardiac arrest, adrenaline [epinephrine] dosage) and a different laboratory parameter (pH) on ICU admission [12]. The severity-of-illness scores APACHE II (Acute Physiology and Chronic Health Evaluation II) and SAPS II (Simplified Acute Physiology Score II) have been widely used in critical care research, and the required clinical and laboratory parameters are readily available for all post-cardiac-arrest patients [24, 25]. All four scores were successfully validated for the prediction of short-term neurological outcomes and mortality [26–35]. However, these scores have not been evaluated regarding long-term outcomes.

This study aims to evaluate the performance of the OHCA, CAHP, APACHE II, and SAPS II scores for early prognostication of long-term mortality and long-term neurological outcome in a large-scale prospective cohort of cardiac arrest patients.

## Methods

### Study setting

This study was conducted using the prospective COMMUNICATE/PROPHETIC cohort of consecutive cardiac arrest patients admitted to the 42-bed interdisciplinary ICU of the University Hospital Basel, Switzerland (tertiary teaching hospital). The COMMUNICATE/PROPHETIC study investigates the outcomes of cardiac arrest patients and the psychosocial stress of their relatives. The details of the study conductance have been published previously [29, 36–41]. Informed consent was either obtained from the patient or the relatives, depending on the decision-making capability of the index patient. In cases of missing relatives, permission was obtained from an independent physician not involved in the study. The study was approved by the Ethics Committee of North-western and Central Switzerland (www.eknz.ch) and followed the principles of the Declaration of Helsinki and its amendments. Analysis and reporting for this study were conducted in accordance with the Transparent Reporting of a Multivariable Prediction Model for Individual Prognosis or Diagnosis (TRIPOD) statement [42].

### Participants

Between October 2012 and November 2019, all patients with ROSC admitted to the ICU after OHCA or in-hospital cardiac arrest (IHCA) were prospectively included. Not eligible were patients suffering a cardiac arrest while being monitored (ICU, intermediate care unit, operating theatre, cardiac catheterisation laboratory). Further exclusion criteria were age  < 16 years or denial of informed consent. The patients were treated according to the standardised local treatment protocol, including targeted temperature management, in compliance with the guidelines of the European Resuscitation Council [8, 43, 44].

### Outcomes

The primary outcome was long-term mortality at 2 years. Secondary outcomes were long-term neurological outcome at 2 years assessed by the Cerebral Performance Category (CPC), and long-term mortality at 6 years. The CPC system was used by the original development studies [11, 12] and most validation studies in accordance with the Utstein Style of reporting data from OHCA [45]. It divides neurological outcome into five categories: CPC = 1: good cerebral performance; CPC = 2: moderate cerebral disability; CPC = 3: severe cerebral disability; CPC = 4: coma or vegetative state; CPC = 5: death or brain death. Good neurological outcome was defined as a CPC of 1 or 2, and poor neurological outcome as a CPC of 3 to 5 in accordance with the development studies [11, 12].

### Data collection

The clinical information was prospectively collected from patient records by the study team. For the calculations of the respective scores, the methodologies of the original publications were strictly applied [11, 12, 24, 25]. Information on resuscitation parameters was collected, including no-flow and low-flow interval, setting of cardiac arrest, initial rhythm, drugs administered, and whether bystander basic life support was performed, as well as clinical data (e. g., heart rate, blood pressure, respiratory rate, urine output, temperature, Glasgow Coma Scale [GCS], intubation status), demographic data (age, sex), pre-existing medical conditions (hypertension, coronary artery disease, congestive heart failure, chronic obstructive pulmonary disease, diabetes, chronic kidney and liver disease, malignant disease) and blood parameters (pH, lactate, base excess, bicarbonate, creatinine, urea, sodium, potassium, bilirubin). Predictor data for calculation of the scores were complete in 79.0% (OHCA), 67.2% (CAHP), 82.9% (APACHE II), and 84.6% (SAPS II), respectively. Missing values were primarily the no-flow time (missing in 12.8%), which is necessary for calculating the OHCA and CAHP scores, and the initial pH (missing in 14.0%), which is required for the CAHP score only. To account for missing data to calculate the four scores, imputed datasets using multiple imputations by chained equations were used for comparisons between scores. Imputations were calculated using multiple covariables (i.e., socio-demographics, comorbidities, resuscitation information, vital signs) also including main outcomes (death, neurological outcome) to reduce bias as previously suggested [46].

### Follow-up and survival

In the context of the COMMUNICATE/PROPHETIC study, all patients who had consented to be contacted by the study team were scheduled for a standardised telephone follow-up after 2 years with an assessment of vital status and neurological performance. After the 2-year follow-up period, survival data were obtained by directly contacting either the patient, their relatives, or their general practitioner. If patient contact was not possible, the medical records of the University Hospital Basel (Switzerland) and publicly available death registries were consulted for information concerning vital status. Patients lost to follow-up were censored at the date of the last follow-up. The follow-up time was calculated from the moment of admission to death or censoring date, whichever date came first.

### Score risk categories

According to the original publication of the CAHP score [12] and a validation study of the OHCA score [26], the CAHP score results were divided into three categories (< 150; 150–200; > 200 points), and the OHCA score results into four categories (≤ 20; > 20–40; > 40–60; > 60 points). Higher risk score categories are associated with a higher risk of an unfavourable outcome [12, 26]. For the APACHE II and the SAPS II scores, corresponding risk categories do not exist [24, 25].

### Statistical analysis

For continuous variables, descriptive statistics such as means, medians, and interquartile ranges were used, and categorical or binary variables were analysed by counts and proportions. Binary and categorical variables between groups were compared using Pearson’s *χ*^2^-test. Continuous data were checked graphically for normality of the distribution. Continuous, normally distributed variables were compared using analysis of variance (ANOVA) or *t*-test, and continuous, skewed variables were compared using the Wilcoxon rank-sum test. The scores were calculated according to the original publications [11, 12, 24, 25]. To assess the prognostic performance of the scores, measures of discrimination and calibration were calculated. Discriminatory performance was summarised by the area under the receiver operating curve (AUROC) for all endpoints. An AUROC of 0.7–0.8 was classified as acceptable, 0.8–0.9 as good, and  > 0.9 as excellent. The approach suggested by DeLong et al. was used to compare ROC curves between groups or between scores [47]. For the OHCA and CAHP score, sensitivity, specificity, positive predictive value (PPV), negative predictive value (NPV), positive and negative likelihood ratio were calculated for the cut-offs described above. The association of the score value with the outcomes was assessed by conducting regression analyses with calculation of hazard ratios (HR) and their 95% confidence intervals (CI) for mortality using Cox-regression models for time to event data (i.e., mortality) and logistic regression analyses with odds ratios (OR) for poor neurological outcome. Calibration of the OHCA and CAHP score was assessed graphically by depicting observed vs. expected outcome event numbers per decile of predicted risk on a calibration plot. All statistical analyses were conducted using STATA 15 (Stata Corporation, College Station, United States of America).

## Results

### Baseline characteristics

During the study period, 486 cardiac arrest survivors were admitted to the ICU of the University Hospital Basel, Switzerland. The final analysis included 415 patients, as 38 patients were excluded due to withheld informed consent or screening failure, and 33 patients were lost to follow-up before assessment of vital status at 2 years. Secondary outcome data were available for 80.2% (CPC at 2 years) and 70.4% (6-year mortality) of the included patients. Baseline characteristics stratified by the primary endpoint of 2-year survival are presented in Table 1. Factors significantly associated with higher mortality were female gender, higher age, chronic comorbidities (chronic obstructive pulmonary disease, diabetes, chronic kidney disease, cancer), longer no-flow and low-flow intervals, unwitnessed cardiac arrest, and non-shockable initial rhythm, as well as lower GCS score, higher lactate levels and lower pH on ICU admission.

**Table 1:** Baseline characteristics

	All	2-year survivors	2-year non-survivors	*p*-value
n	415	174 (41.9%)	241 (58.1%)	
Sociodemographics				
Age (years), mean (SD)	64.7 (14.9)	59 (14.5)	68.8 (13.7)	< 0.001
Male gender	301 (72.5%)	141 (81.0%)	160 (66.4%)	< 0.001
Comorbidities				
Hypertension	206 (49.9%)	85 (48.9%)	121 (50.6%)	0.72
Coronary artery disease	242 (58.6%)	114 (65.5%)	128 (53.6%)	0.015
Congestive heart failure	58 (14.0%)	20 (11.5%)	38 (15.9%)	0.2
COPD	40 (9.7%)	5 (2.9%)	35 (14.6%)	< 0.001
Diabetes	91 (22.0%)	24 (13.8%)	67 (28.0%)	< 0.001
Chronic kidney disease	61 (14.8%)	14 (8.0%)	47 (19.7%)	0.001
End-stage liver disease	9 (2.2%)	1 (0.6%)	8 (3.3%)	0.057
Malignant disease	47 (11.4%)	7 (4.0%)	40 (16.7%)	< 0.001
Resuscitation parameters				
IHCA	54 (13.0%)	20 (11.5%)	34 (14.2%)	0.43
No-flow time (minutes), mean (SD)	4 (6)	1 (3)	5 (7)	< 0.001
Low-flow time (minutes), mean (SD)	20 (17)	16 (13)	23 (19)	< 0.001
Witnessed cardiac arrest	334 (81.1%)	162 (93.6%)	172 (72.0%)	< 0.001
Bystander CPR	280 (67.8%)	145 (83.3%)	135 (56.5%)	< 0.001
Bystander CPR professional	93 (40.8%)	49 (45.0%)	44 (37.0%)	0.22
Initial rhythm				< 0.001
VF	204 (49.4%)	125 (71.8%)	79 (33.1%)	
VT	19 (4.6%)	11 (6.3%)	8 (3.3%)	
PEA	91 (22.0%)	12 (6.9%)	79 (33.1%)	
Asystole	75 (18.2%)	10 (5.7%)	65 (27.2%)	
Unknown	24 (5.8%)	16 (9.2%)	8 (3.3%)	
Clinical/laboratory parameters on ICU admission				
GCS, mean (SD)	5 (4)	7 (5)	4 (3)	< 0.001
Endotracheal intubation	353 (86.1%)	127 (74.3%)	226 (94.6%)	< 0.001
Haemodynamic support (mechanical)	47 (11.5%)	15 (8.8%)	32 (13.4%)	0.15
Haemodynamic support (pharmacological)	289 (70.5%)	111 (64.9%)	178 (74.5%)	0.036
Lactate (mmol/l), mean (SD)	6.8 (4.4)	4.9 (3.1)	8.1 (4.7)	< 0.001
pH, mean (SD)	7.22 (0.16)	7.27 (0.11)	7.20 (0.17)	< 0.001

Data presented as *n* (%) unless otherwise specified

*COPD* chronic obstructive pulmonary disease, *CPR* cardiopulmonary resuscitation, *GCS* Glasgow Coma Scale, *IHCA* in-hospital cardiac arrest, *PEA* pulseless electrical activity, *SD* standard deviation, *VF* ventricular fibrillation, *VT* ventricular tachycardia

### Mortality and neurological outcome

Of 415 patients, 201 (48.4%) died during the initial hospital stay. Withdrawal of life-sustaining therapy was conducted in 179 of these 201 patients (89%). After 2 years, 241 of 415 patients (58.1%) died. Of the 92 survivors with an assessment of neurological outcome after 2 years, 89 (96.7%) had a good neurological outcome (CPC 1–2), and 3 (3.3%) a poor neurological outcome (CPC 3–4). After 6 years, 241 of 292 patients (82.5%) died. A Kaplan–Meier survival estimate of the total cohort is shown in Additional file 1: Figure S1.

### Prognostic performance of risk scores

**Fig. 1:**
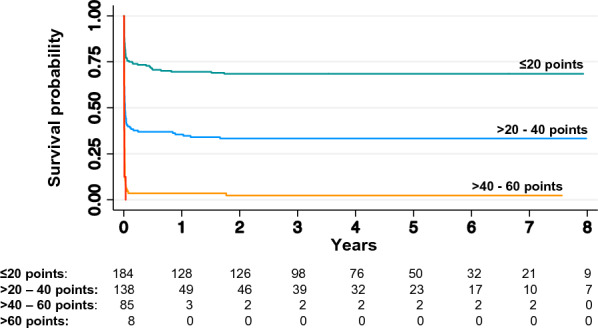
Kaplan–Meier survival estimates with number at risk for predefined OHCA score categories. Below the *x*-axis, number at risk for the individual time points are reported

**Fig. 2:**
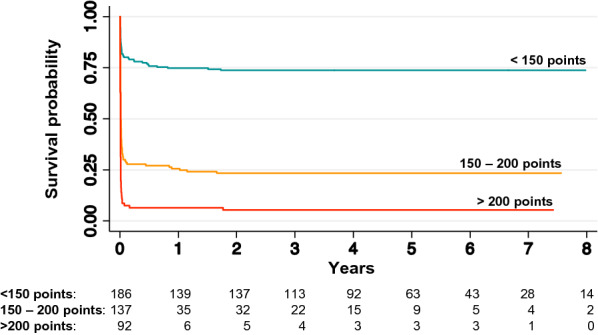
Kaplan–Meier survival estimates with number at risk for predefined CAHP score categories. Below the *x*-axis, number at risk for the individual time points are reported

Table 2 summarises the prognostic performance of the OHCA, CAHP, SAPS II, and APACHE II scores for the prognostication of the primary and secondary outcomes. For 2-year mortality, all scores showed good discriminatory performance, with the CAHP yielding an AUROC of 0.87 (95% CI 0.84–0.90), followed by the APACHE II score (0.83 [95% CI 0.79–0.87]), the OHCA score (0.82 [95% CI 0.78–0.86]) and the SAPS II score (0.81 [95% CI 0.76–0.85]). The differences between the AUROC values were statistically significant (*χ*^2^ = 19.4, *p* < 0.001). A graphical comparison of ROC curves for 2-year mortality is shown in Additional file 1: Figure S2. The CAHP showed good discriminatory performance for the secondary endpoints with an AUROC of 0.86 (95% CI 0.82–0.90) for the 2-year neurological outcome and an AUROC of 0.88 (95% CI 0.83–0.93) for 6-year mortality. All other scores showed acceptable to good discriminatory performance for the secondary endpoints (for details see Table 2). For the OHCA and CAHP score, prognostic accuracy at the predefined cut-offs is presented in Tables 3 and 4, respectively. An OHCA score of  > 40 points predicted 2-year mortality with a specificity of 98.9% (95% CI 95.9–99.9), the highest risk category (> 60 points) reached a specificity of 100% (95% CI 63.1–100.0). The CAHP score’s high-risk category (> 200 points) predicted 2-year mortality with a specificity of 97.1% (95% CI 93.4–99.1). Figures 1 and 2 show Kaplan–Meier survival estimate curves with numbers at risk stratified by OHCA and CAHP score categories. For all endpoints, the AUROC was additionally calculated for the subgroups of OHCA and IHCA patients and the results shown in Additional file 1: Table S1.

**Table 2:** Comparison of long-term prognostic performance between scores

Score	OHCA	CAHP	APACHE II	SAPS II
A: mortality at 2 years				
Score points in all patients (*n* = 415)	23 (8, 38)	161 (118, 196)	30 (26, 35)	65 (55, 75)
Score points in survivors (*n* = 174)	9 (−3, 22)	118.5 (93, 145)	27 (22, 30)	58 (48, 66)
Score points in non-survivors (*n* = 241)	31 (21, 45)	188 (160, 219)	33 (29, 38)	72 (61, 81)
* p*-value (Wilcoxon rank-sum test)	< 0.001	< 0.001	< 0.001	< 0.001
HR per quartile (95% CI)	2.11 (1.85, 2.40)	2.31 (2.02, 2.64)	1.95 (1.73, 2.21)	1.92 (1.70, 2.17)
AUROC (95% CI)	0.82 (0.78, 0.86)	0.87 (0.84, 0.90)	0.83 (0.79, 0.87)	0.81 (0.76, 0.85)
B: mortality at 6 years				
Score points in all patients (*n* = 292)	29 (15, 44)	179 (140, 209)	32 (28, 37)	68 (58, 79)
Score points in survivors (*n* = 51)	13 (−2, 25)	115 (90, 141)	28 (23, 31)	58 (49.5, 67)
Score points in non-survivors (*n* = 241)	31 (21, 45)	188 (160, 219)	33 (29, 38)	72 (61, 81)
* p*-value (Wilcoxon rank-sum test)	< 0.001	< 0.001	< 0.001	< 0.001
HR per quartile (95% CI)	1.56 (1.37, 1.77)	1.79 (1.57, 2.04)	1.61 (1.42, 1.81)	1.55 (1.37, 1.74)
AUROC (95% CI)	0.78 (0.71, 0.84)	0.88 (0.83, 0.93)	0.83 (0.77, 0.90)	0.80 (0.74, 0.87)
C: neurological outcome at 2 years				
Score points in all patients (*n* = 333)	27 (13, 42)	171 (134, 204)	31 (27, 36)	67 (58, 78)
Score points in patients with good neurological outcome (*n* = 89)	12 (−1, 22)	119 (101, 145)	27 (23, 30)	60 (48, 68)
Score points in patients with poor neurological outcome (*n* = 244)	31 (21, 45)	188 (159.5, 218.5)	33 (29, 38)	72 (61, 81)
* p*-value (Wilcoxon rank-sum test)	< 0.001	< 0.001	< 0.001	< 0.001
OR per quartile (95% CI)	2.99 (2.25, 3.98)	3.86 (2.82, 5.30)	3.23 (2.41, 4.32)	2.49 (1.91, 3.24)
AUROC (95% CI)	0.81 (0.76, 0.85)	0.86 (0.82, 0.90)	0.83 (0.78, 0.88)	0.78 (0.73, 0.84)

Data presented as median (IQR) unless otherwise specified

*APACHE II* Acute Physiology and Chronic Health Evaluation Score II, *AUROC* area under the receiver operating characteristics curve, *CAHP* Cardiac Arrest Hospital Prognosis Score, *OHCA* Out-of-Hospital Cardiac Arrest Score, *HR* hazard ratio, *OR* odds ratio, *SAPS II* Simplified Acute Physiology Score II

**Table 3:** Performance of OHCA score at different cut-off points

OHCA category	> I	> II	> III
	Cut-off 20 points	Cut-off 40 points	Cut-off 60 points
A: mortality at 2 years			
Total number of patients *n*	231	93	8
2-year survivors *n* (%)	48 (20.8)	2 (2.1)	0 (0)
Death within 2 years *n* (%)	183 (79.2)	91 (97.9)	8 (100)
Sensitivity	75.9 (70.0, 81.2)	37.8 (31.6, 44.2)	3.3 (1.4, 6.4)
Specificity	72.4 (65.1, 78.9)	98.9 (95.9, 99.9)	100.0 (97.9, 100.0)
PPV	79.2 (73.4, 84.3)	97.8 (92.4, 99.7)	100.0 (63.1, 100.0)
NPV	68.5 (61.2, 75.1)	53.4 (47.8, 59.0)	42.8 (37.9, 47.7)
LLR +	2.75 (2.14, 3.54)	32.85 (8.20, 131.55)	n. a
LLR−	0.33 (0.26, 0.42)	0.63 (0.57, 0.70)	0.97 (0.94, 0.99)
B: mortality at 6 years			
Total number of patients *n*	202	93	8
6-year survivors *n* (%)	19 (9.4)	2 (2.1)	0 (0)
Death within 6 years *n* (%)	183 (90.6)	91 (97.9)	8 (100)
Sensitivity	75.9 (70.0, 81.2)	37.8 (31.6, 44.2)	3.3 (1.4, 6.4)
Specificity	62.7 (48.1, 75.9)	96.1 (86.5, 99.5)	100.0 (93.0, 100.0)
PPV	90.6 (85.7, 94.2)	97.8 (92.4, 99.7)	100.0 (63.1, 100.0)
NPV	35.6 (25.7, 46.3)	24.6 (18.8, 31.2)	18.0 (13.7, 22.9)
LLR +	2.04 (1.42, 2.93)	9.63 (2.45, 37.82)	n. a
LLR−	0.38 (0.28, 0.52)	0.65 (0.58, 0.73)	0.97 (0.94, 0.99)
C: neurological outcome at 2 years			
Total number of patients *n*	212	93	8
Good neurological outcome *n* (%)	27 (12.7)	2 (2.1)	0 (0)
Poor neurological outcome *n* (%)	185 (87.3)	91 (97.9)	8 (100)
Sensitivity	75.8 (69.9, 81.1)	37.3 (31.2, 43.7)	3.3 (1.4, 6.4)
Specificity	69.7 (59.0, 79.0)	97.8 (92.1, 99.7)	100.0 (95.9, 100.0)
PPV	87.3 (82.0, 91.4)	97.8 (92.4, 99.7)	100.0 (63.1, 100.0)
NPV	51.2 (42.0, 60.4)	36.3 (30.2, 42.7)	27.4 (22.6, 32.6)
LLR +	2.50 (1.81, 3.45)	16.60 (4.18, 65.96)	n. a
LLR−	0.35 (0.27, 0.45)	0.64 (0.58, 0.71)	0.97 (0.95, 0.99)

Data presented as mean (95% CI) unless otherwise specified

*LLR* + positive likelihood ratio, *LLR−* negative likelihood ratio, *NPV* negative predictive value, *OHCA* Out-of-Hospital Cardiac Arrest Score, *PPV* positive predictive value

**Table 4:** Performance of CAHP-Score at different cut-off points

CAHP category	> I	> II
	Cut-off 150 points	Cut-off 200 points
A: mortality at 2 years		
Total number of patients *n*	229	92
2-year survivors *n* (%)	37 (16.2)	5 (5.4)
Death within 2 years *n* (%)	192 (83.8)	87 (94.6)
Sensitivity	79.7 (74.0, 84.6)	36.1 (30.0, 42.5)
Specificity	78.7 (71.9, 84.6)	97.1 (93.4, 99.1)
PPV	83.8 (78.4, 88.4)	94.6 (87.8, 98.2)
NPV	73.7 (66.7, 79.8)	52.3 (46.7, 57.9)
LLR +	3.75 (2.80, 5.02)	12.56 (5.21, 30.29)
LLR−	0.26 (0.20, 0.34)	0.66 (0.60, 0.73)
B: mortality at 6 years		
Total number of patients *n*	200	90
6-year survivors *n* (%)	8 (4.0)	3 (3.3)
Death within 6 years *n* (%)	192 (96.0)	87 (96.7)
Sensitivity	79.7 (74.0, 84.6)	36.1 (30.0, 42.5)
Specificity	84.3 (71.4, 93.0)	94.1 (83.8, 98.8)
PPV	96.0 (92.3, 98.3)	96.7 (90.6, 99.3)
NPV	46.7 (36.3, 57.4)	23.8 (18.1, 30.2)
LLR +	5.08 (2.68, 9.63)	6.14 (2.02, 18.63)
LLR−	0.24 (0.18, 0.32)	0.68 (0.60, 0.76)
C: neurological outcome at 2 years		
Total number of patients *n*	213	91
Good neurological outcome *n* (%)	20 (9.4)	3 (3.3)
Poor neurological outcome *n* (%)	193 (90.6)	88 (96.7)
Sensitivity	79.1 (73.5, 84.0)	36.1 (30.0, 42.4)
Specificity	77.5 (67.4, 85.7)	96.6 (90.5, 99.3)
PPV	90.6 (85.9, 94.2)	96.7 (90.7, 99.3)
NPV	57.5 (48.1, 66.5)	35.5 (29.5, 41.9)
LLR +	3.52 (2.38, 5.21)	10.70 (3.47, 32.95)
LLR−	0.27 (0.21, 0.35)	0.66 (0.60, 0.73)

Data presented as mean (95% CI) unless otherwise specified

*LLR* + positive likelihood ratio, *LLR−* negative likelihood ratio, *NPV* negative predictive value, *OHCA* Out-of-Hospital Cardiac Arrest Score, *PPV* positive predictive value

The CAHP score showed good calibration for 2-year mortality with a slight overestimation of mortality in the upper two-thirds of the risk spectrum (Additional file 1: Figure S3). Calibration of the OHCA score was poor, with underestimation of 2-year mortality in the low-risk spectrum and overestimation in the high-risk spectrum (Additional file 1: Figure S4). For 2-year neurological outcome, the calibration of the CAHP score was good, with a slight underestimation of poor neurological outcome, especially in the lower risk categories (Additional file 1: Figure S5). The OHCA score showed poor calibration for 2-year neurological outcome with underestimation in the low-risk spectrum and overestimation in the higher risk spectrum (Additional file 1: Figure S6).

## Discussion

This study has validated two prognostic cardiac arrest scores (OHCA and CAHP scores), and two ICU severity-of-illness scores (APACHE II and SAPS II scores) for the prediction of long-term mortality and neurological outcome in a prospective cohort of cardiac arrest survivors followed for up to 8 years. The CAHP score showed the best discriminatory performance for the prediction of 2-year mortality, 6-year mortality, and 2-year neurological outcome. Calibration of the CAHP score was good for 2-year mortality and 2-year neurological outcome. As already demonstrated for the prognostication of short-term outcomes [29], two non-specific severity-of-illness scores, the APACHE II and the SAPS II showed promising discriminatory performance for the prognostication of long-term survival as well as long-term neurological outcome. The main drawback of the APACHE II and the SAPS II score is that the worst value of the first 24 h after ICU admission is required for each included parameter. This results in a time delay compared with the OHCA and CAHP scores, which require only parameters readily available on ICU admission.

The findings of this study are generally in line with previous validation studies evaluating outcomes at hospital discharge or 30 days post-event, where the CAHP score showed a slightly better performance than the OHCA score [29, 30, 33, 48]. In two different cohorts of cardiac arrest patients evaluating outcomes at hospital discharge [49] or 90 days [32] the OHCA score performed somewhat better than the CAHP score. The severity-of-illness scores had a slightly inferior performance when compared to the cardiac arrest-specific scores, which was also noted in previous studies looking at short-term outcomes [29, 50, 51]. A British group recently developed and validated a post-cardiac arrest score for OHCA patients to predict neurological outcome 6 months after OHCA and compared it with the OHCA and CAHP scores [52]. Their score only showed marginally better discrimination when compared with the CAHP score in their cohort (AUROC 0.88 vs. 0.87, respectively) [52]. One may argue that the development of new scores for the prognostication of long-term outcomes may not provide additional value, as established scores perform well in predicting long-term outcomes. Efforts to improve established scoring systems by adding known predictors of outcome after cardiac arrest, such as laboratory parameters (e. g., neuron-specific enolase), imaging or electrophysiological examination results, or clinical signs (e. g., GCS motor score) have shown promising results [30, 32, 40]. Such modifications with corresponding validation studies might be helpful to keep established scores up to date and improve their predictive value based on current and evolving science.

A major and overarching concern in research regarding prognostic factors in post-cardiac-arrest patients is the effect of self-fulfilling prophecy, meaning that the documentation of poor prognosis early in the treatment process per se may lead to a change or withdrawal of care, which again leads to a higher occurrence of poor outcome in this patient group [53–55]. In our study, score values were calculated by the study team and were not provided to the treating ICU physicians, thus minimising the risk of a low score value influencing the ICU team in their decision-making. However, treating physicians inevitably knew about different clinical factors which also have been used for calculating the score values (e. g., no-flow and low-flow intervals, laboratory values). These factors might have influenced their decision-making. However, blinding involved clinicians with respect to these factors is not possible.

The presented data suggest that the established cardiac arrest-specific scores OHCA and CAHP, which have been thoroughly validated for predicting short-term mortality and neurological outcome in OHCA and IHCA patients, might be suitable for predicting long-term outcomes. Although the OHCA and CAHP scores have originally been intended for the prognostication of outcomes after OHCA, both scores have since been successfully validated in OHCA and IHCA survivors [29, 56, 57], which is confirmed by the results of the present study. However, a subgroup analysis showed significantly lower discriminatory performance of both the OHCA and CAHP scores when used for the prediction of 2-year mortality in IHCA patients only compared to OHCA patients. This finding is in line with previous studies and was expected, as the scores were originally developed for use in OHCA patients only. Before applying the scores to IHCA patients, further validations and, if necessary, adaptions and/or recalibration of the scores are recommended.

The OHCA and CAHP score can easily be calculated using openly accessible online calculators, rendering their use easy and straightforward [58, 59]. Further randomised controlled trials using the scores as decision aids are needed to test the impact of prediction models on decision-making, outcomes, and healthcare costs in clinical practice. In addition, validation studies based on other long-term cohorts of cardiac arrest patients are needed to further validate and, if necessary, recalibrate the scores for the prognostication of long-term outcomes.

Our study has limitations. First, we did not have complete data for all parameters to calculate the scores and thus had to impute the missing data. Second, due to the study setting, there was a relatively large proportion of loss of follow-up patients resulting in a possible selection bias. This is mainly the case for the secondary outcomes. Assessment of neurological outcome at 2 years of follow-up required a telephone interview, which some patients declined while others could not be contacted by the study team. As a 6-year follow-up was not part of the original study design, a substantial proportion of patients were lost to follow-up. Therefore, the results for 2-year neurological outcome and 6-year mortality have to be interpreted with caution due to a possible selection bias. Third, we only assessed all-cause mortality and thus patients may have died from other unrelated causes. Fourth, the single-centre setting of the study limits the generalisability of the results to other centres, regions or countries. External validation studies evaluating the herein validated scores for long-term outcomes in other populations are necessary to address this issue. However, the relatively large cohort size of the study, the fact that treatment modalities were in line with other Swiss and European medical centres, and the inclusion of unselected cardiac arrest patients indicate a high external validity of the results. Furthermore, analysis and reporting were conducted according to current state-of-the-art methodological guidelines, so that its results can be of the greatest possible use for future research in this field.

## Conclusion

In our single-centre cohort of cardiac arrest survivors, the OHCA, CAHP, APACHE II, and SAPS II scores showed good performance in early prognostication of long-term mortality at 2 years and acceptable to good performance for the prognostication of 6-year mortality and neurological outcome at 2 years after cardiac arrest. Of the herein validated scoring systems, the CAHP score showed the best discriminatory performance and is a simple-to-use risk-stratification tool available early after cardiac arrest. These scores thus may guide clinicians by stratifying patients according to the risk of poor long-term outcome and may help to support discussions about goals-of-care and the extent of therapeutic effort.

## Supplementary Information

**Additional file 1: Table S1.** Subgroup analysis. **Figure S1.** Kaplan Meier survival estimate for the entire cohort. Number at risk for the individual time points are reported. **Figure S2.** Comparison of ROC curves for the primary outcome 2-year mortality. **Figure S3.** Calibration plot depicting observed vs. expected numbers of primary outcome (2-year mortality) per decile of risk as predicted by the CAHP score. **Figure S4.** Calibration plot depicting observed vs. expected numbers of primary outcome (2-year mortality) per decile of risk as predicted by the OHCA score. **Figure S5.** Calibration plot depicting observed vs. expected numbers of poor neurological outcome at 2 years per decile of risk as predicted by the CAHP score. **Figure S6.** Calibration plot depicting observed vs. expected numbers of poor neurological outcome at 2 years per decile of risk as predicted by the OHCA score.

## Data Availability

The datasets generated and/or analysed during the current study are available from the corresponding author on reasonable request.

## Abbreviations

APACHE II: Acute Physiology and Chronic Health Evaluation II
AUROC: Area under the receiver operating characteristics curve
CAHP: Cardiac Arrest Hospital Prognosis
CI: Confidence interval
CPC: Cerebral performance category
GCS: Glasgow Coma Scale
ICU: Intensive care unit
IHCA: In-hospital cardiac arrest
NPV: Negative predictive value
OHCA: Out-of-hospital cardiac arrest
PPV: Positive predictive value
ROSC: Return of spontaneous circulation
SAPS II: Simplified Acute Physiology Score II
TRIPOD: Transparent reporting of a multivariable prediction model for individual prognosis or diagnosis
