# Supplementary material for: Comparison of different clinical risk scores to predict long-term survival and neurological outcome in adults after cardiac arrest: results from a prospective cohort study
Source: Ann Intensive Care. 2022 Aug 17;12:77. doi: 10.1186/s13613-022-01048-y (PMC9385915; doi:10.1186/s13613-022-01048-y)
Supplement: Supplementary file 1 — Additional file 1: Table S1. Subgroup analysis. Figure S1. Kaplan Meier survival estimate for the entire cohort. Number at risk for the individual time points are reported. Figure S2. Comparison of ROC curves for the primary outcome 2-year mortality. Figure S3. Calibration plot depicting observed vs. expected numbers of primary outcome (2-year mortality) per decile of risk as predicted by the CAHP score. Figure S4. Calibration plot depicting observed vs. expected numbers of primary outcome (2-year mortality) per decile of risk as predicted by the OHCA score. Figure S5. Calibration plot depicting observed vs. expected numbers of poor neurological outcome at 2 years per decile of risk as predicted by the CAHP score. Figure S6. Calibration plot depicting observed vs. expected numbers of poor neurological outcome at 2 years per decile of risk as predicted by the OHCA score. [file 13613_2022_1048_MOESM1_ESM.pdf]

Supplementary Material

**Supplementary Table 1 – Subgroup analysis**				
**Score**	**Subgroup**	**AUROC**		
		2-year mortality	2-year neurological outcome	6-year mortality
OHCA	OHCA	0.85 (0.81-0.89)	0.83 (0.78-0.88)	0.80 (0.74-0.87)
	IHCA	0.68 (0.53-0.83)	0.74 (0.54-0.93)	0.69 (0.36-1.00)
	p-value	0.0277	0.3519	0.5290
CAHP	OHCA	0.89 (0.86-0.92)	0.88 (0.84-0.92)	0.89 (0.83-0.94)
	IHCA	0.74 (0.60-0.88)	0.69 (0.46-0.92)	0.89 (0.71-1.00)
	p-value	0.0353	0.1152	0.9751
APACHE II	OHCA	0.84 (0.80-0.88)	0.84 (0.79-0.89)	0.84 (0.77-0.90)
	IHCA	0.76 (0.62-0.90)	0.70 (0.41-0.98)	0.80 (0.48-1.00)
	p-value	0.2720	0.3337	0.8108
SAPS II	OHCA	0.82 (0.77-0.85)	0.79 (0.74-0.84)	0.79 (0.73-0.86)
	IHCA	0.75 (0.61-0.88)	0.72 (0.43-1.00)	0.88 (0.70-1.00)
	p-value	0.3553	0.6355	0.4053
Score performance in the subgroups of OHCA patients and IHCA patients. Statistical comparison between AUROC was conducted according to DeLong et al. (1988), p-values are from the X^2-test. **APACHE II** Acute Physiology and Chronic Health Evaluation Score II; **AUROC** Area under the receiver operating characteristics curve; **CAHP** Cardiac Arrest Hospital Prognosis Score; **IHCA** In-Hospital Cardiac Arrest; **OHCA** Out-of-Hospital Cardiac Arrest Score; **SAPS II** Simplified Acute Physiology Score II.				

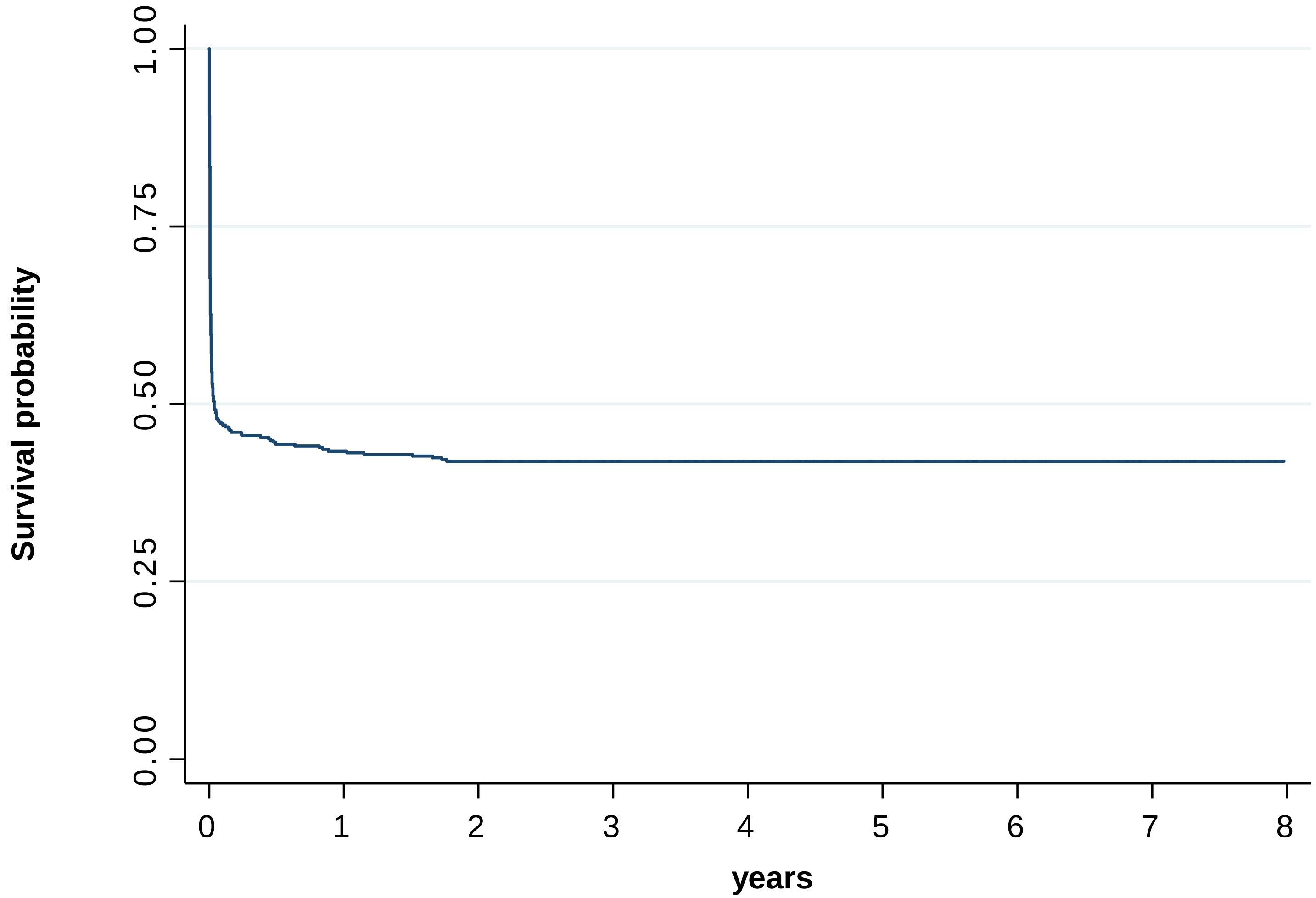

Number at risk 415 180 174 139 110 75 51 33 16

Supplementary Figure 1. Kaplan Meier survival estimate for the entire cohort. Number at risk for the individual time points are reported.

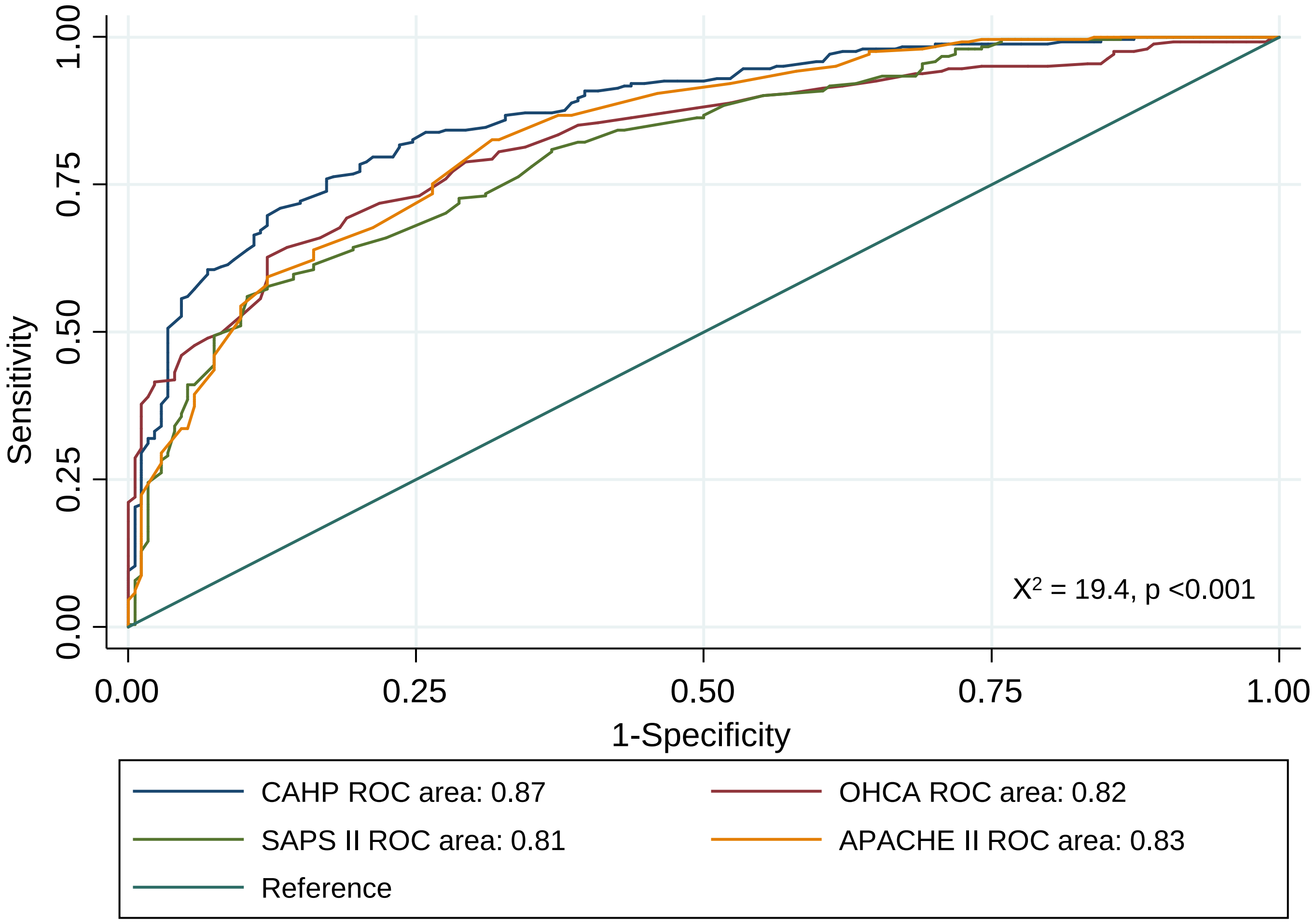

Supplementary Figure 2. Comparison of ROC curves for the primary outcome 2-year mortality.

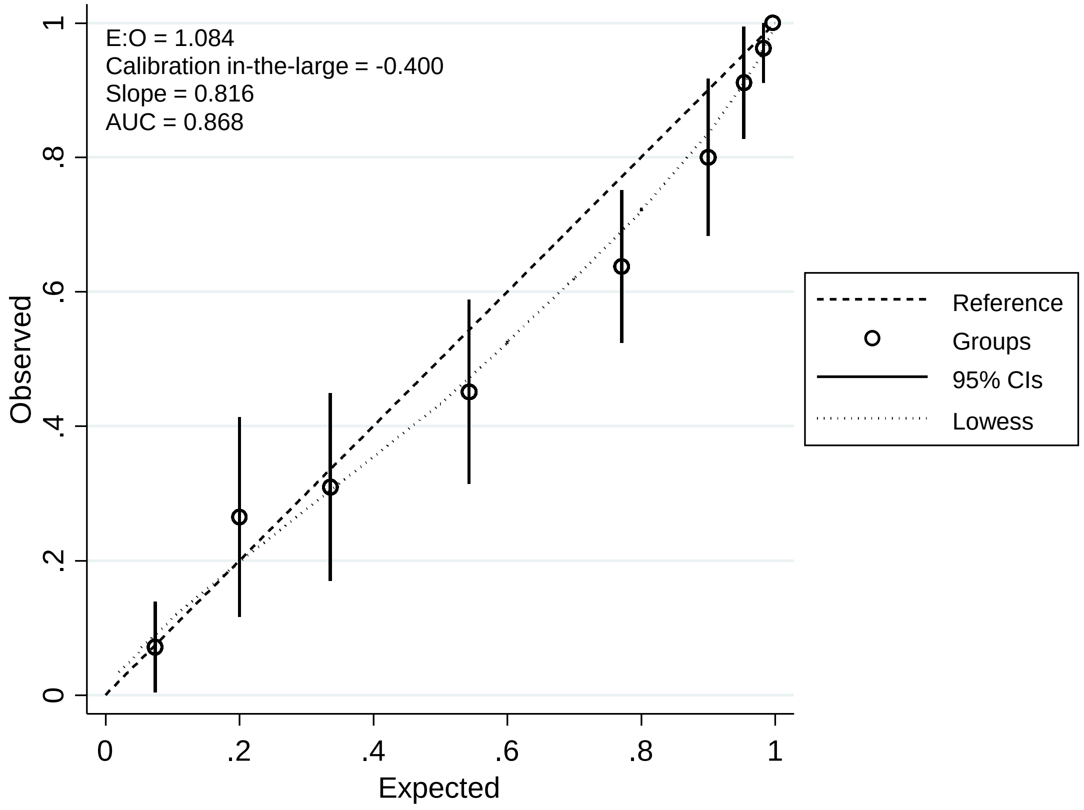

Supplementary Figure 3. Calibration plot depicting observed vs. expected numbers of primary outcome (2-year mortality) per decile of risk as predicted by the CAHP score.

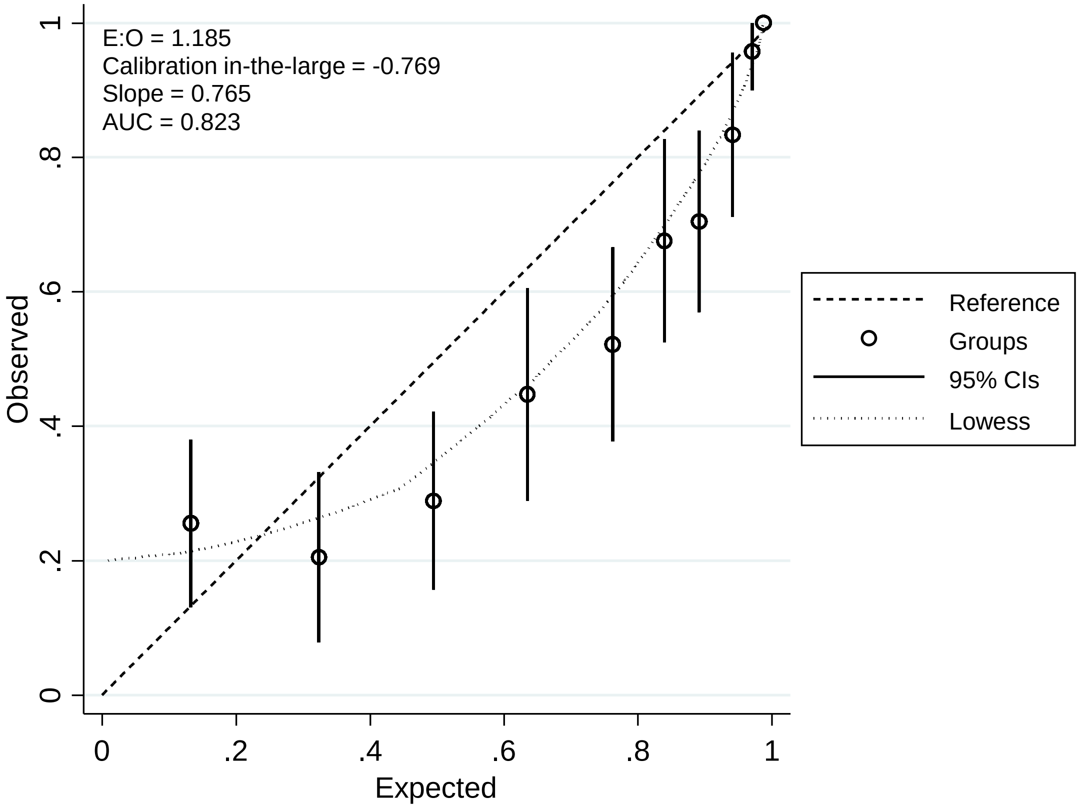

Supplementary Figure 4. Calibration plot depicting observed vs. expected numbers of primary outcome (2-year mortality) per decile of risk as predicted by the OHCA score.

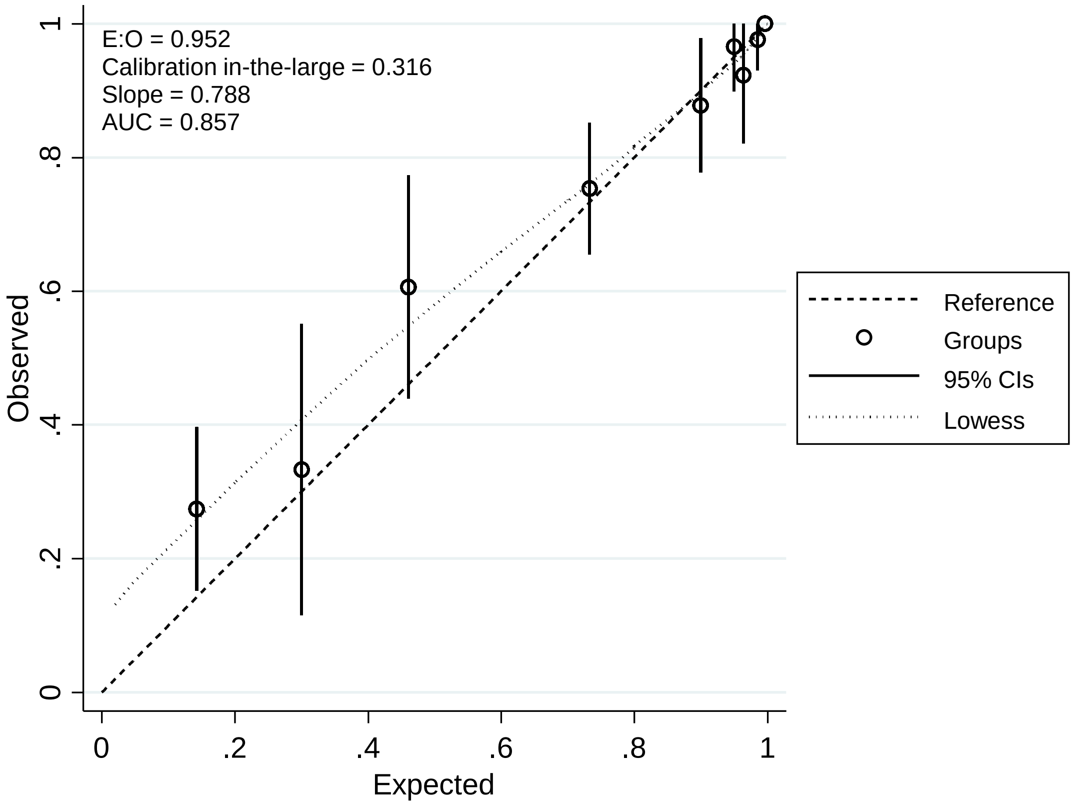

Supplementary Figure 5. Calibration plot depicting observed vs. expected numbers of poor neurological outcome at 2 years per decile of risk as predicted by the CAHP score.

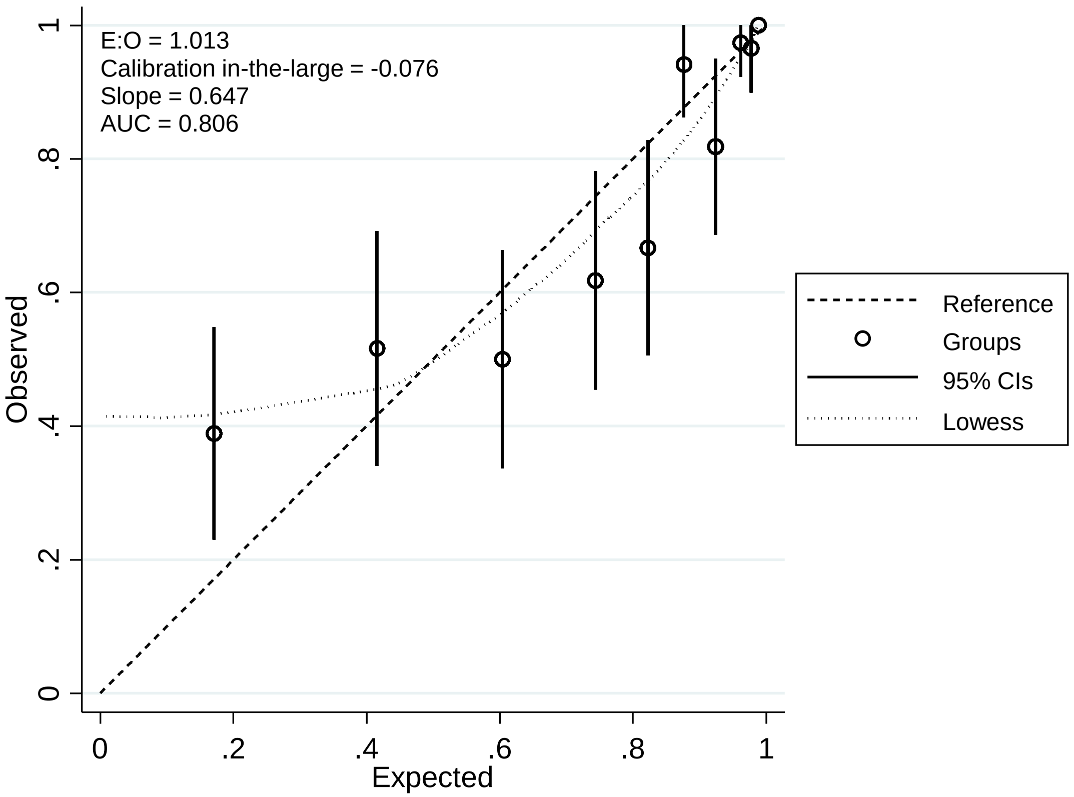

Supplementary Figure 6. Calibration plot depicting observed vs. expected numbers of poor neurological outcome at 2 years per decile of risk as predicted by the OHCA score.
